# The atorvastatin metabolic phenotype shift is influenced by interaction of drug-transporter polymorphisms in Mexican population: results of a randomized trial

**DOI:** 10.1038/s41598-020-65843-y

**Published:** 2020-06-01

**Authors:** Rafael B. R. León-Cachón, Aileen-Diane Bamford, Irene Meester, Hugo Alberto Barrera-Saldaña, Magdalena Gómez-Silva, María F. García Bustos

**Affiliations:** 1 Center of Molecular Diagnostics and Personalized Medicine, Department of Basic Sciences, Division of Health Sciences, University of Monterrey, San Pedro Garza Garcia, Nuevo Leon Mexico; 2 Vitagenesis S.A., Monterrey, Nuevo Leon Mexico; 3 Innbiogem S.C., Monterrey, Nuevo Leon Mexico; 4 Forensic Medicine Service, School of Medicine, Autonomous University of Nuevo Leon, Monterrey, Nuevo Leon Mexico; 5 Analytical Department of the Research Institute for Clinical and Experimental Pharmacology, Ipharma S.A., Monterrey, Nuevo Leon Mexico; 6 Institute of Experimental Pathology (CONICET), Faculty of Health Sciences, National University of Salta, Salta, Argentina; 7 University School in Health Sciences, Catholic University of Salta, Salta, Argentina

**Keywords:** Cardiology, Molecular medicine, Genetics, Genetic association study, Genetic interaction, Genetic markers, Haplotypes, Population genetics

## Abstract

Atorvastatin (ATV) is a blood cholesterol-lowering drug used to prevent cardiovascular events, the leading cause of death worldwide. As pharmacokinetics, metabolism and response vary among individuals, we wanted to determine the most reliable metabolic ATV phenotypes and identify novel and preponderant genetic markers that affect ATV plasma levels. A controlled, randomized, crossover, single-blind, three-treatment, three-period, and six-sequence clinical study of ATV (single 80-mg oral dose) was conducted among 60 healthy Mexican men. ATV plasma levels were measured using high-performance liquid chromatography mass spectrometry. Genotyping was performed by real-time PCR with TaqMan probes. Four ATV metabolizer phenotypes were found: slow, intermediate, normal and fast. Six gene polymorphisms, *SLCO1B1*-rs4149056, *ABCB1*-rs1045642, *CYP2D6*-rs1135840, *CYP2B6*-rs3745274, *NAT2*-rs1208, and *COMT*- rs4680, had a significant effect on ATV pharmacokinetics (*P* < 0.05). The polymorphisms in *SLCO1B1* and *ABCB1* seemed to have a greater effect and were especially important for the shift from an intermediate to a normal metabolizer. This is the first study that demonstrates how the interaction of genetic variants affect metabolic phenotyping and improves understanding of how *SLCO1B1* and *ABCB1* variants that affect statin metabolism may partially explain the variability in drug response. Notwithstanding, the influence of other genetic and non-genetic factors is not ruled out.

## Introduction

Cardiovascular diseases (CVD) are the leading cause of death worldwide^1^ and in Mexico^2^. Diet, a lack of physical activity, and ageing are risk factors for CVD, but smoking, a high blood pressure and a high blood cholesterol level are at the top^3^. Statins are the first-choice drugs to treat hypercholesterolemia, and atorvastatin (ATV) is one of the most used statins^4,5^. However, interindividual variability in both ATV metabolism^6–8^ and therapeutic response^9–11^ have been reported. Three ATV metabolic phenotypes have been identified^6,8,12^ and non-validated massive genotyping methods identified genetic variants that seemed to impact ATV pharmacokinetics^6,13^.

The classification of metabolic phenotypes is challenging because of large datasets, variables at different scales, and limited knowledge on phenotyping data management^14^. Gene expression studies on large data sets of tissue samples^15^ and patients^16^ often apply cluster analysis, but its use is uncommon in pharmacogenomics and pharmacogenetics^6,17^. Cluster analysis of pharmacogenetics data could facilitate the screening for possible pharmacokinetic and metabolic profiles if the optimal number of groups and cut-off limits can be defined.

Several genes have been related to the variability in statin metabolism and response. Hepatic uptake and clearance largely depend on influx and efflux transporters such as those encoded by the genes *ABCB1* and *SLCO1B1*^7,18^. In biotransformation, genes encoding phase I metabolic enzymes, such as *CYP3A4*, *CYP3A5*, *CYP2D6*, and *CYP2B6*, are relevant, because they metabolize many drugs. Although it is known that *CYP3A4* and *CYP3A5* metabolize statins, the polymorphisms *CYP3A4*-rs2740574 and *CYP3A5*-rs776746 occurred at a low frequency in the Mexican population and no effect on ATV metabolism was detected. The contribution of *CYP2D6* variants could not be tested due to a low call rate of the genotyping method. A potential effect of *CYP2B6* has not been confirmed. Likewise, the effect on ATV metabolism could not be proven for variants of genes encoding phase II metabolic enzymes (*NAT2* and *COMT*) for the same reasons^6,13,19^. In this study among a Mexican population, we applied a novel approach to identify and confirm ATV metabolic phenotypes and associated pharmacogenetic profiles. Hereto, we selected candidate genes involved in the metabolism and response to drugs, *i.e. ABCB1, SLCO1B1* and *CYP2D6*, based on previous reports, frequency, and importance in the Mexican population and analyzed them under genetic models to identify or confirm their effect on the pharmacokinetics of ATV.

## Results

### Study population

All participants were healthy non-related male Mexicans who identified themselves as mestizos. Most (93.1%) were residents from northeastern Mexican states, namely Nuevo Leon (83.3%), Coahuila (1.6%), Tamaulipas (3.3%) and San Luis Potosi (4.9%). The volunteers had similar anthropometric data, and no significant differences in body composition. No adverse effects due to drug administration occurred^6^.

**Figure 1:**
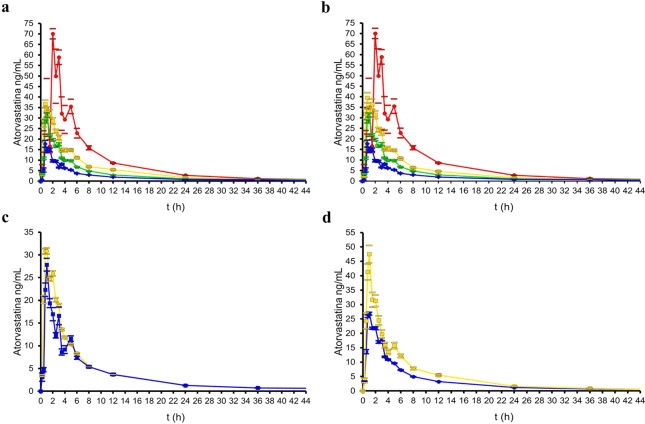
Mean peak plasma ATV concentration-time curves, according to phenotypes and genotypes. (**a)** Pharmacokinetic profiles of different metabolic phenotypes. (**b)** Pharmacokinetic profiles of different metabolic phenotypes with *ABCB1*-rs1045642 genotype C/C adjusting. For both (**a**,**b**): slow metabolizers (*red*), intermediate metabolizers (*orange*), normal metabolizers (*green*) and fast metabolizers (*blue*). (**c)** Pharmacokinetic profiles of *ABCB1*-rs1045642 genotypes: carriers of C/C genotype (*blue*) and carriers of C/T or T/T genotypes (*orange*) (**d)** Pharmacokinetic profiles of *SLCO1B1*-rs4149056 genotypes: carriers of C/C or C/T genotype (*blue*) and carriers of T/T genotypes (*orange*). Mean peak plasma concentration-time curves after single 80-mg dose of ATV. Data shown are mean ± standard error (SE) concentrations.

### Pharmacokinetic analysis

Under controlled conditions, the mean ± standard deviation (SD) of adjusted pharmacokinetic parameters used for phenotyping were: maximum plasma concentration (C_max_) = 41.70 ± 21.51 ng/mL, the area under the plasma ATV concentration-time curve (AUC) from time 0 to the time of last measurement (AUC_0-t_) = 143.35 ± 84.64 ng/mL*h, AUC from time 0 extrapolated to infinity (AUC_0-∞_) = 155.84 ± 85.79 ng/mL*h, and the total drug clearance (Cl) = 0.65 ± 0.31 L/h*kg (Table 1). The coefficient of variation (CV) was greater than 50% for all parameters, except for Cl, which was 48%.

**Table 1:** Pharmacokinetic parameters and cut-off according to metabolizer phenotype.

Phenotypes	n	Pharmacokinetic parameters			
		C_max_ (ng/ml)^a^	AUC_0-t_ (ng/ml*h)^b^	AUC_0-∞_ (ng/ml*h)^b^	Cl (L/h*kg)^b^
Slow	3	87.05 ± 3.29	411.91 ± 49.18	421.06 ± 49.24	0.19 ± 0.02
		(78.88-95.22)	(289.74–534.07)	(298.73–543.38)	(0.14–0.25)
Intermediate	15	54.50 ± 20.40	206.49 ± 45.95	223.19 ± 48.57	0.37 ± 0.07
		(43.20–65.79)	(181.04–231.94)	(196.29–250.08)	(0.33–0.41)
Normal	28	39.20 ± 13.46	119.73 ± 17.41	130.94 ± 18.59	0.62 ± 0.09
		(33.98–44.42)	(112.98–126.48)	(123.73–138.15)	(0.59–0.66)
Fast	14	21.95 ± 9.00	65.37 ± 15.52	76.66 ± 14.7	1.09 ± 0.26
		(16.76–27.15)	(56.41–74.33)	(68.17–85.15)	(0.94–1.24)
All subjects	60	41.70 ± 21.51	143.35 ± 84.64	155.84 ± 85.79	0.65 ± 0.31

Data shown as mean ± standard deviation, cut-off limits in parenthesis. ^a^P ≤ 1.6×10–4 for comparison between all phenotypes, except intermediate vs. normal (P = 0.094); ^b^P ≤ 0.002 for comparison between all phenotypes. Cmax, maximum plasma concentration; AUC, area under the plasma concentration-time curve; AUC0-t, AUC from time 0 to the time of last measurement; AUC0- ∞ , AUC from time 0 extrapolated to infinity; Cl, total clearance.

### ATV metabolizer phenotypes

Cluster analysis distinguished four clusters or phenotypes that most reliably explained the observed pharmacokinetic variability (Table 1). When we applied this algorithm, the adjusted R-square for C_max_ was 0.519; for AUC_0-t_, 0.865; for AUC_0-∞_, 0.866; and for Cl, 0.783 (*P* < 0.05). The defined phenotypes were: slow metabolizers (n = 3), intermediate metabolizers (n = 15), normal metabolizers (n = 28) and fast metabolizers (n = 14) as shown in Fig. 1a. Aforementioned pharmacokinetic parameters were significantly different (*P* < 0.05) among the metabolizer phenotypes, except for the C_max_ values between intermediate and normal metabolizers (*P* = 0.09). Mean ATV pharmacokinetic parameters differed more than 10-fold between the fastest and slowest metabolizer groups (C_max_ = 8.80 ng/mL, AUC_0-t_ = 38.42 ng/mL*h, AUC_0-∞_ = 46.92 ng/mL*h, Cl = 1.71 L/h*kg *vs*. C_max_ = 89.86 ng/mL, AUC_0-t_ = 467.93 ng/mL*h, AUC_0-∞_ = 477.81 ng/mL*h and Cl = 0.17 L/h*kg; respectively).

### Pharmacogenetic tests

The call rate for the seven gene polymorphisms was 1.0. All genetic markers were in Hardy-Weinberg Equilibrium (*P* > 0.05) and had a minor allele frequency > 0.01. The haplotype analysis revealed that the most frequent haplotypes were CTTT (n = 26; 43%) CCTT (n = 12; 20%) and TTTT (n = 11; 18%), result of the combination of *ABCB1*-rs1045642 and *SLCO1B1*-rs4149056 polymorphisms.

### Effect of gene polymorphisms and haplotypes on atorvastatin pharmacokinetics

Five gene polymorphisms had a significant effect on different pharmacokinetic parameters. C_max_ values were influenced by *ABCB1*-rs1045642 polymorphism under a dominant model. C/C carriers had a significant lower C_max_ (*P* = 0.038) than C/T and T/T carriers (Table 2). With respect to *SLCO1B1*-rs4149056, C carriers (homozygous or heterozygous) had a higher AUC_0-t_, AUC_0-∞_, Cl and volume of distribution (V_d_) than the homozygous T genotype (*P* ≤ 0.038; Table 2). Regarding the metabolizer enzyme variants, heterozygous carriers (C/G) of *CYP2D6*-rs1135840 had higher elimination rate constant in the terminal drug phase (Ke) values, but significantly lower half-life (T_1/2_) values than those with homozygous genotypes (*P* ≤ 0.022; Table 2). Similarly, *COMT*-rs4680 G/G carriers had a higher AUC_0-∞_, but lower Cl than A/A and A/G carriers (*P* ≤ 0.048; Table 2). We also found a significant effect of the CCTT haplotype (of the combination of *ABCB1* and *SLCO1B1* drug transporters) on C_max_, AUC_0-t_, AUC_0-∞_ and Cl parameters (*P* ≤ 0.040). Subjects with a CCTT genetic profile in drug transporter genes had lower C_max_, AUC_0-t_, and AUC_0-∞_, but a higher Cl than those with other combinations (Table 2). The regression analysis confirmed that three gene polymorphisms (*ABCB1*-rs1045642, *SLCO1B1*-rs4149056 and *COMT*-rs4680) had an impact on ATV pharmacokinetics (Table 3). The regression analysis also revealed that *CYP2B6*-rs3745274 and *NAT2*-rs1208 are involved in pharmacokinetic variability. The *CYP2B6*-rs3745274 polymorphisms influenced the variability of C_max_ (in the over-dominant model; Table 3) and *NAT2*-rs1208 variants affect AUC_0-∞_ (under the co-dominant and dominant models; Table 3). No other polymorphisms had a significant effect on ATV pharmacokinetics.

**Table 2:** Effect of gene variants on ATV pharmacokinetics.

Genotypes	n	C_max_ (ng/ml)	AUC_0-t_ (ng/ml*h)	AUC_0-∞_ (ng/ml*h)	K_e_	T_1/2_ (h)	Cl (L/h*kg)	V_d_ (L/kg)
***ABCB1-*rs1045642**								
C/C	14	31.22 ± 15.38	115.33 ± 64.68	127.6 ± 65.25	0.0871 ± 0.0488	10.33 ± 5.20	0.81 ± 0.45	10.61 ± 4.68
C/T	32	45.76 ± 21.51	159.45 ± 100.64	173.28 ± 102.13	0.0760 ± 0.0290	11.62 ± 7.69	0.59 ± 0.26	9.2 ± 5.77
T/T	14	41.59 ± 22.49	134.56 ± 51.09	144.22 ± 50.17	0.0759 ± 0.0314	10.94 ± 5.19	0.62 ± 0.20	9.39 ± 4.70
C/T + T/T	46	44.49 ± 21.64^a^	151.87 ± 88.69	164.44 ± 89.97	0.0760 ± 0.0294	11.41 ± 6.98	0.60 ± 0.24	9.26 ± 5.42
***SLCO1B1*-rs4149056**								
C/C	1	47.00 ± N.A.	198.79 ± N.A.	288.28 ± N.A.	0.0190 ± N.A.	36.43 ± N.A.	0.28 ± N.A.	14.58 ± N.A.
C/T	10	50.90 ± 27.37	210.16 ± 112.11	220.93 ± 112.54	0.0774 ± 0.0294	10.02 ± 3.40	0.45 ± 0.22	5.90 ± 2.20
T/T	49	39.34 ± 19.45	128.58 ± 72.33	139.86 ± 71.85	0.08000 ± 0.0353	10.88 ± 6.11	0.70 ± 0.31	10.22 ± 5.40
C/C + C/T	11	50.55 ± 25.99	209.13 ± 106.41^b^	227.05 ± 108.68^c^	0.0721 ± 0.0330	12.42 ± 8.59	0.43 ± 0.21^d^	6.68 ± 3.35^e^
***CYP2B6*-rs3745274**								
G/G	36	46.30 ± 23.54	156.06 ± 99.43	167.17 ± 98.65	0.0808 ± 0.0355	10.42 ± 5.29	0.62 ± 0.29	8.82 ± 5.08
G/T	22	33.72 ± 14.57	123.39 ± 52.47	138.82 ± 60.93	0.0732 ± 0.0349	12.75 ± 8.35	0.70 ± 0.36	11.08 ± 5.46
T/T	2	37.39 ± 0.18	133.99 ± 63.54	139.23 ± 63.96	0.0982 ± 0.0059	7.07 ± 0.42	0.64 ± 0.30	6.46 ± 2.62
G/G + T/T	38	45.83 ± 22.98	154.9 ± 97.40	165.7 ± 96.73	0.0817 ± 0.0348	10.24 ± 5.20	0.62 ± 0.28	8.70 ± 4.99
**CYP2D6-rs16947**								
A/A	3	35.31 ± 10.03	135.17 ± 40.13	155.20 ± 26.99	0.0582 ± 0.0335	15.21 ± 9.12	0.52 ± 0.09	12.28 ± 8.99
A/G	24	42.80 ± 24.14	133.18 ± 88.41	145.03 ± 87.42	0.0859 ± 0.0413	10.64 ± 6.96	0.72 ± 0.38	10.27 ± 6.35
G/G	33	40.92 ± 19.59	151.48 ± 85.65	163.76 ± 88.76	0.0751 ± 0.0290	11.18 ± 6.17	0.61 ± 0.27	8.82 ± 3.88
A/A + G/G	36	40.45 ± 18.95	150.13 ± 82.59	163.05 ± 85.15	0.0737 ± 0.0292	11.51 ± 6.39	0.60 ± 0.26	9.11 ± 4.40
***CYP2D6*-rs1135840**								
C/C	25	42.15 ± 20.49	155.64 ± 90.39	169.10 ± 94.13	0.0714 ± 0.0300	11.97 ± 6.74	0.60 ± 0.27	9.22 ± 4.19
C/G	28	39.65 ± 21.74	126.68 ± 74.86	137.21 ± 73.28	0.0896 ± 0.0376	9.55 ± 5.52	0.73 ± 0.35	9.41 ± 5.46
G/G	7	45.65 ± 22.29	166.11 ± 100.07	183.04 ± 97.68	0.0600 ± 0.0268	14.74 ± 8.77	0.53 ± 0.23	11.48 ± 7.86
C/C + G/G	32	42.91 ± 20.58	157.93 ± 91.01	172.15 ± 93.49	0.0689 ± 0.0293^f^	12.57 ± 7.17^g^	0.58 ± 0.26	9.72 ± 5.14
***NAT2*-rs1208**								
A/A	25	46.39 ± 22.25	156.10 ± 110.43	171.21 ± 112.16	0.0827 ± 0.0444	11.98 ± 8.53	0.64 ± 0.35	9.76 ± 5.91
A/G	28	37.81 ± 19.58	135.79 ± 64.94	146.88 ± 64.75	0.0741 ± 0.0235	10.70 ± 4.94	0.66 ± 0.31	9.69 ± 5.24
G/G	7	37.89 ± 21.38	128.01 ± 38.24	136.80 ± 37.38	0.0818 ± 0.0365	10.08 ± 4.45	0.63 ± 0.19	8.41 ± 2.49
A/G + G/G	35	37.82 ± 19.62	134.24 ± 60.14	144.87 ± 59.94	0.0756 ± 0.0261	10.58 ± 4.79	0.65 ± 0.29	9.44 ± 4.81
***COMT*-rs4680**								
A/A	9	30.47 ± 9.09	107.08 ± 33.40	116.03 ± 36.04	0.0859 ± 0.0363	9.69 ± 4.59	0.74 ± 0.20	9.60 ± 3.37
A/G	29	39.67 ± 21.27	139.81 ± 93.84	152.93 ± 96.83	0.0794 ± 0.0293	10.93 ± 7.04	0.69 ± 0.36	9.71 ± 5.17
G/G	22	48.13 ± 22.42	162.85 ± 83.54	175.97 ± 81.02	0.0744 ± 0.0413	12.07 ± 6.76	0.55 ± 0.26	9.37 ± 6.11
A/A + A/G	38	37.49 ± 19.39	132.06 ± 84.29	144.19 ± 87.34^h^	0.0810 ± 0.0307	10.64 ± 6.51	0.70 ± 0.33^i^	9.69 ± 4.76

Data shown as mean ± standard deviation. ^a^P = 0.038 (C/C vs. C/T + T/T); ^b^P = 0.003 (T/T vs. C/C + C/T); ^c^P = 0.004 (T/T vs. C/C + C/T); ^d^P = 0.005 (T/T vs. C/C + C/T); ^e^P = 0.038; ^f^P = 0.020 (C/G vs. C/C + G/G); ^g^P = 0.022 (C/G vs. C/C + G/G); ^h^P = 0.046 (A/A + A/G vs. G/G); ^i^ P = 0.048 (A/A + A/G vs. G/G). Cmax, maximum plasma concentration; AUC, area under the plasma concentration-time curve; AUC0-t, AUC from time 0 to the time of last measurement; AUC0- ∞ , AUC from time 0 extrapolated to infinity; Ke, elimination rate constant in the terminal drug phase; T1/2, half-life drug; Cl, total clearance; Vd, volume of distribution. N.A., not apply.

**Table 3:** Pharmacokinetic parameters of gene transporters haplotype.

Phenotypes	n	Pharmacokinetic parameters						
		C_max_ (ng/ml)	AUC_0-t_ (ng/ml*h)	AUC_0-∞_ (ng/ml*h)	K_e_	T_1/2_ (h)	Cl (L/h*kg)	V_d_ (L/kg)
CTTT	26	44.95 ± 20.94	143.54 ± 88.13	154.99 ± 87.15	0.0785 ± 0.0286	10.97 ± 6.82	0.64 ± 0.26	9.70 ± 6.05
CCTT	12	30.63 ± 16.58^a^	97.58 ± 45.55^b^	110.08 ± 47.10^c^	0.0921 ± 0.0509	9.88 ± 5.33	0.89 ± 0.44^d^	11.18 ± 4.64
TTTT	11	35.57 ± 15.13	127.03 ± 40.91	136.57 ± 41.00	0.0704 ± 0.0284	11.74 ± 5.48	0.63 ± 0.18	10.39 ± 4.82
CTCT	5	49.74 ± 28.62	234.28 ± 144.62	245.40 ± 143.26	0.0745 ± 0.0235	10.02 ± 2.96	0.42 ± 0.20	5.49 ± 1.91
TTCT	3	63.63 ± 34.83	162.17 ± 84.53	172.25 ± 80.35	0.0960 ± 0.0402	8.02 ± 2.91	0.56 ± 0.32	5.73 ± 1.48
CCCT	2	34.71 ± 4.62	221.86 ± 71.26	232.78 ± 71.62	0.0569 ± 0.0205	13.03 ± 4.69	0.36 ± 0.11	7.15 ± 4.53
CTCC	1	47.00 ± N.A.	198 ± N.A.	288.28 ± N.A.	0.0190 ± N.A.	36.43 ± N.A.	0.28 ± N.A.	14.58 ± N.A.
Not-CCTT	48	44.08 ± 21.28	154.79 ± 88.53	167.28 ± 89.73	0.0752 ± 0.0292	11.48 ± 6.87	0.59 ± 0.24	9.17 ± 5.36

Data shown as mean ± standard deviation. ^a^P = 0.040 (CCTT vs. Not-CCTT); ^b^P = 0.028 (CCTT vs. Not-CCTT); ^c^P = 0.037 (CCTT vs. Not-CCTT); ^d^P = 0.040 (CCTT vs. Not-CCTT). P values supported by linear, logistic and logarithmic regression analysis. Cmax, maximum plasma concentration; AUC, area under the plasma concentration-time curve; AUC0-t, AUC from time 0 to the time of last measurement; AUC0- ∞ , AUC from time 0 extrapolated to infinity; Ke, elimination rate constant in the terminal drug phase; T1/2, half-life drug; Cl, total clearance; Vd, volume of distribution. N.A., not apply.

### Interaction of gene polymorphism on atorvastatin pharmacokinetics

The interaction of *ABCB1*-rs1045642 with *CYP2B6*-rs3745274, under the dominant and over-dominant model, respectively, predicted 11.1% of the C_max_ variability (Table 3). Furthermore, under the co-dominant model, the interaction of *SLCO1B1*-rs4149056 with *NAT2*-rs1208 predicted 19.7% of the AUC_0-∞_ variation. In another approach to test gene polymorphism interactions, after a genotype adjustment of intermediate metabolizers, *i.e*. three subjects with a C/C genotype for *ABCB1*-rs1045642 were removed from the group, the normal and intermediate metabolizers became significantly different (Fig. 1b). The new mean ± SD for C_max_ of intermediate metabolizers became 59.23 ± 20.12 ng/mL (cut-off 46.45–72.02 ng/mL) and the adjusted R-square increased to 0.700 (*P* < 0.005). In addition, CCTT carriers were only ones that were statistically different from other subjects (Table 4).

**Table 4:** Gene predictors models of the pharmacokinetic parameters of atorvastatin.

Parameter	Model	Predictors	Genetic model	R square	Adjusted R square	P-Value
C_max_	1	rs4680	Co-dominant	0.083	0.067	0.026^a^
	2	rs3745274	Over-dominant	0.079	0.063	0.030^a^
	3	rs3745274, rs1045642	Over-dominant, dominant	0.141	0.111	0.046^a^
AUC_0-t_	1	rs4149056	Co-dominant	0.124	0.109	0.006^a^
	2	rs4149056	Recessive	0.138	0.123	0.003^a^
AUC_0-∞_	1	rs4149056	Co-dominant	0.166	0.152	0.001^a^
	2	rs4149056, rs1208	Co-dominant	0.225	0.197	0.043^b^
	3	rs4149056	Recessive	0.157	0.143	0.002^a^
	4	rs4149056, rs1208	Recessive, dominant	0.219	0.192	0.038^b^
Cl	1	rs4149056	Co-dominant	0.113	0.097	0.009^a^

^a^P-value supported by logistic, logarithmic and linear regression analysis. ^b^ P-value supported by linear regression. *C*_*max*_, maximum plasma concentration; *AUC*, area under the plasma concentration-time curve; *AUC*_*0-t*_, AUC from time 0 to the time of last measurement; *AUC*_*0-∞*_, AUC from time 0 extrapolated to infinity; *Cl*, total clearance.

### Phenotype-genotype association

When the association between phenotypes and genotypes was assessed, we found that, under the recessive model, *SLCO1B1*-rs4149056 is associated with fast/normal metabolizers while the presence of allele C is related to slow/intermediate metabolizers (Table 5). This association remained after Bonferroni correction and was supported by logistic regression (*P* = 0.002). No association was found between other haplotypes and phenotypes.

**Table 5:** Association between genotypes and metabolizer phenotypes.

Gene	Polymorphism	Model	OR (95% CI)	P-Value	Pc-Value
*SLCO1B1*	rs4149056	Recessive (T/T vs. C/C + C/T)	T/T: Fast/normal metabolizers	0.001	0.002*
			0.10 (0.02–0.43)		
			C/C + C/T: Intermediate/slow metabolizers		
			10.40 (2.33–46.51)		

*OR*, odds ratio; *CI*, confidence interval; *Pc*, Bonferroni-corrected P-values. *Pc value supported by logistic regression.

## Discussion

Despite similar health conditions, body composition, and controlled experimental conditions, ATV pharmacokinetic parameters varied greatly among Mexican male individuals. We applied a novel approach (cluster analysis based on more pharmacokinetic parameters than a previous method) that distinguished four metabolizer phenotypes with higher accuracy and reliability than a previously reported classification method^6^. Furthermore, it allowed the selection of a model with the highest prediction percentage and best cut-off limits for each metabolizer phenotype. In addition, the analysis allowed identifying inconsistencies. For example, after phenotyping, considerable variability of C_max_ values was observed in the intermediate metabolizer group, suggesting that there are important differences between individuals in the absorption process.

Although the clustering analysis method has been successfully applied for classification purposes in other areas of biomedical research^14–16^, it has been little used in pharmacokinetics, pharmacogenetics and pharmacogenomics^6^. A reason might be the need to realize multiple analyzes to select the ideal number of clusters. In our experience, the cluster analysis method proved to be a) effective in identifying the different groups within a population, b) useful to determine which are the groups or subgroups that require more focus, and c) practical to evaluate the absorption, distribution, metabolism and excretion (ADME) process when no metabolic data are available, as only pharmacokinetic data are required.

The classification for this Mexican population should not be extrapolated indiscriminately to other populations, because population-specific intrinsic genetic variation may shift pharmacokinetics. Thus, population-specific ATV pharmacokinetic stratification criteria should be determined. Hereto, we recommend to develop an analogous cluster analysis which can be applied retrospectively. The pharmacokinetic variability of ATV observed in our population differed from the one reported for a Bengali population^20^. In both studies, the volunteers were young adult males, but the dose (40 *vs*. 80 mg) and race (Bengali *vs*. Mexican genetic background) differed between the studies.

We assessed common genetic variants involved in drug metabolism to verify and confirm their influence on ATV pharmacokinetic variability. After genotyping validation, the genotype distribution of *ABCB1*-rs1045642 (0.23 for C/C; 0.53 for C/T and 0.23 for T/T) differed from a previously reported distribution that had been obtained with microarrays^6^. Importantly, the effect on C_max_ values remains significant. The *ABCB1* gene encodes a transporter protein that has affinity for multiple substrates, both endogenous and exogenous ones^21^. The polymorphism rs1045642 is located in exon 26 and produces a synonymous substitution, so the role of the *ABCB1* variant in plasma drug concentrations is controversial^22^. Even so, rs1045642 variant may influence the folding time of the protein, alter its specificity, and therefore influence the concentration of a given drug^23^. In addition, synergistic or antagonistic interactions with other gene variants may create variability. So far, evidence on synergistic or antagonistic effect of *ABCB1* on ATV plasma concentrations is scarce^13^.The significantly lower values in C/C carriers and the dominant effect of allele T suggest that the rs1045642 polymorphism significantly affects the absorption, bioavailability and blood concentrations of ATV. This hypothesis is supported by the intestinal expression of *ABCB1*, where this transporter actively participates in drug absorption^24^. These data on rs1045642 are consistent with previous findings, even for different drugs and populations. T/T carriers had a higher C_max_ for ATV among Americans and for rosuvastatin among Chinese, while C/C carriers had a lower C_max_ and AUC for edoxaban and also in Mexicans for amfepramone^17,18,25,26^. Contrasting data, however, were found in a Korean population, where T/T carriers had a lower C_max_ and longer half-life than C allele carriers. The group size was small (n = 3), though^27^. As far as we know, there is no other report about the effect of *ABCB1*-rs1045642 on the pharmacokinetics of other statins^28,29^. Our findings suggest that a lower concentration of drug may yield a lower response, whereas an increased exposure to the medicine could cause adverse effects. Conversely, the lack of pharmacodynamic data did not allow us to evaluate the response to treatment and confirm our hypothesis. Despite this limitation, the association between *ABCB1*-rs1045642 and the pharmacological response to ATV has been well documented. For example, the C/C genotype among Australian patients treated with ATV associated with a lower treatment efficiency (*i.e*. less decrease in LDL values) as compared to other genotypes^30^. Similar data have been reported for Egyptian males^9^, Iranian^31^ and Polish populations^32^, and in a meta-analysis where 395 patients were included and treated with statins^33^. Regarding adverse effects, a higher frequency of the T allele has been found in patients who presented with myalgia, but no association has been reported^30^.

In our analysis, the genotype frequency of *SLCO1B1*-rs4149056 was similar to the one we have previously reported^6^. The new clustering analysis method confirmed the effect of *SLCO1B1*-rs4149056 on ATV pharmacokinetics. The *SLCO1B1* gene codes for a protein responsible for the transport of organic anions and other compounds, such as drugs. This gene is expressed exclusively in the liver, where it has an important role in metabolism^34^. The rs4149056 polymorphism generates a p.V174A substitution that causes a decrease in expression and transport activity^35^. *SLCO1B1*-rs4149056 had the greatest effect on ATV pharmacokinetics. The presence of the C allele in *SLCO1B1*-rs4149056 seemed to affect AUC_0-t_, AUC_0-∞_, Cl, V_d_, and therefore impact the exposure to ATV, which was consistent with its occurrence in normal/faster phenotypes. The association between *SLCO1B1*-rs4149056 and aforementioned pharmacokinetic parameters indicates that the variant mainly affects the metabolism and excretion phases of ATV in the Mexican population as opposed to *ABCB1*-rs1045642, which apparently affects the absorption phase. This assumption is consistent with ATV pharmacokinetics in Chinese (n = 32), Japanese (n = 31), Caucasian (n = 30), and Korean (n = 28) populations where the presence of the C allele of polymorphism rs4149056 was associated with higher C_max_ and/or AUC values^7,27,36^. In our study, rs4149056 has a preponderant role on ATV metabolism, since it was the only one that showed an association with metabolic phenotypes. Again, the higher AUC and slower clearance in C allele carriers could result in a better response or a higher susceptibility to adverse effects. The latter, may be explained by the statin response studies, where the C allele has been related to the risk of myopathy^37,38^. In all studies, the C/C genotype was the least frequent.

*CYP2D6* codes for one of the main drug metabolizing enzymes, since it participates in the biotransformation of around 25% of all drugs^39^. The *CYP2D6*-rs1135840 polymorphism creates an alternative splicing site that eliminates exon 6, without a significant impact on gene expression^40^. Another *CYP2D6* polymorphism, *CYP2D6*-rs3892097, has been associated with ATV-induced adverse effects on muscle^41^. However, there is little information about the role of *CYP2D6* in ATV metabolism or pharmacokinetics. In a previous study, *CYP2D6*-rs1135840 seemed to have a significant effect on AUC values^13^, but this finding was not confirmed in this study. Here, we identified that *CYP2D6*-rs1135840 has a significant effect on Ke and T_1/2_ parameters, but this was not confirmed with the regression analyses, so its effect on the metabolism and excretion phases is not clear.

The catechol-*O*-methyltransferase, encoded by the *COMT* gene, is an enzyme that helps to eliminate endogenous or toxic metabolites, as well as exogenous polycyclic compounds^42,43^. Due to its regulatory function of catecholamines, more is known about its role in pharmacodynamics than its role in pharmacokinetics^44^. The A allele related to susceptibility to coronary artery disease^44^. We discovered a significant effect of the *COMT*-rs4680 polymorphism on the C_max_, AUC_0-∞_ and Cl of ATV, although this effect was not supported by regression analysis. The rs4680 variant produces a non-synonymous amino acid change (p.V158M) causing impaired COMT activity^44,45^. As far as we know this is the first study that reports a possible effect of *COMT*-rs4680 on ATV pharmacokinetics.

*CYP2B6* is a P450 family pharmacogene responsible for the metabolism of 4% of the main drugs^19,46^. The *CYP2B6* gene is expressed primarily in the liver^19,47^ and its expression can be induced by different substrates including ATV^48^. The rs3745274 is located in exon 4 and produces a p.Q172H substitution, which is related with a slight reduction in expression and activity^19^. The T allele variant of rs3745274 has been related to a lower propofol dose^49^ and an increased exposure to efavirenz^50^. Although ATV induced *CYP2B6* expression in cultured human hepatocytes^51^, the role of *CYP2B6* on ATV pharmacokinetics *in vivo* is unknown. Our results show that, under an over-dominant model, rs3745274 affects the variation of C_max_, suggesting a slight role in ATV absorption of an unknown mechanism.

The N-acetyltransferase 2 gene (*NAT2*) encodes a phase II metabolic enzyme involved in the biotransformation of drugs and carcinogens. *NAT2* has many variations and has been associated with different metabolic phenotypes^52^, especially for anti-tuberculosis drugs^53^. The *NAT2*-rs1208 produces a p.K268R substitution that associates with a rapid acetylation^52^. To date, the influence of *NAT2*-rs1208 on statin pharmacokinetics has not been reported. We found that *NAT2*-rs1208 affected AUC_0-∞_ under co-dominant and dominant models.

Although the effect of *ABCB1* and *SLCO1B1* transporters on pharmacokinetics and pharmacodynamics, as well as on susceptibility to adverse effects has been well documented, there are few studies that assess the interaction of these genes and their relationship with the effectiveness of treatment. Pharmacogenetics data from 1844 subjects suggest that *ABCB1* and *SLCO1B1* variants may be useful for improving effectiveness and preventing the risks of adverse effects of statin treatment^10^. Our data on a Mexican population confirm that the transporters ABCB1 and SLCO1B1 have a significant impact on ATV metabolism (Fig. 1c,d). Although the SLCO1B1 transporter had the greatest impact on ATV metabolism, the influence of ABCB1 is underscored by the fact that the presence of the *ABCB1*-rs1045642 C/C variant is sufficient to shift from an intermediate to a normal metabolism phenotype. Thus, different combinations of these two variants may generate a broad spectrum of metabolism and therefore a variable response to treatment. In our knowledge, this is the first study that demonstrates how the interaction of genetic variants affect metabolic phenotyping and improves understanding of how SLCO1B1 and ABCB1 variants that affect statin metabolism may explain the variability in drug response.

In the present study, some limitations remain. First, as only men were included in this study, our findings should be confirmed in women. Second, the lack of data on secondary metabolites of ATV did not allow us to validate the metabolic classification by another method. Third, this study was limited to candidate polymorphisms. Thus, other polymorphisms or genes that could have an impact on the metabolism of ATV were not investigated.

## Conclusions

Variants of the transporter-encoding genes *ABCB1* and *SLCO1B1* have an important impact on ATV pharmacokinetics in a Mexican male population. Hence, the metabolism also varies from population to population, even for the same drug. So, it is important that each study perform its metabolic classification. Our results improve the understanding of the mechanism by which variation in transporters may affect the therapeutic response to ATV in a Mexican population. *ABCB1* and *SLCO1B1* variants were not only congruent with, but could also explain, the metabolic phenotype classification at a genetic level.

## Material and methods

### Design

This pharmacogenetic study used pharmacokinetic data from a controlled, randomized, crossover, single-blind, three-treatment, three-period, and six-sequence clinical study after a single 80-mg oral dose of ATV (tablets; Pfizer Pharmaceuticals LLC, Caguas Site, Caguas, PR), conducted in 60 healthy Mexican men. The clinical protocol complied with national and international ethical regulations, guidelines and norms, as described previously^6^, and was performed under medical supervision. The clinical protocol was approved by the Research and Ethics Committee of the Clinical and Experimental Pharmacology Center, Ipharma S. A. (Monterrey, NL, MEX), and registered at the Federal Commission for Protection Against Health Risks (COFEPRIS code: Atorvastatina/A95-10Bis) and the Australian New Zealand Clinical Trials Registry (ACTRN12614000851662, registration date: 08/08/2014). The pharmacogenetic procedure was approved by the Ethics, Research and Biosecurity Committees of the University of Monterrey (San Pedro Garza Garcia, NL, MEX; registry number 042014-CIE). Volunteers provided a written informed consent.

### Study population

Between January and February 2011, sixty healthy, 18-to-45-year-old, non-smoking, Mexican males with a weight ≥ 50 kg and a body mass index between 20 and 26 kg/m^2^ participated in the bioequivalence study. Their health status was assessed based on physical examination, medical history, and clinical and biochemical tests. Exclusion criteria included abnormal laboratory results, drug abuse, ingestion of alcohol 1 week prior to the study, the use of medication three weeks before enrollment, and participation in a clinical research study within the previous 3 months.

### Dosing regimen and sampling

The dosing and sampling involved a three-treatment (R = reference drug, T1 = Test 1 and T2 = Test 2 drugs are first and second treatment, respectively), three-period, and six-sequence schedule with a 2-week washout period between treatments. Before starting any drug administration, the statistical department of Ipharma S.A. randomized drug allocation (R, T1 or T2, file code, and subject code) with a balanced design using the Mersenne Twister algorithm and R statistical software. The participants were blinded to treatment. About 18 h before the first dose administration, the subjects were admitted to the clinical site and served a standard dinner (<800 kcal). The next morning, after an overnight fast, ATV was administered orally as a single 80-mg dose tablet (Pfizer, New York, NY, USA) with 240 mL of water. A standardized lunch was served 4 h after dosing and standardized dinner at 12 h after dosing. Peripheral blood (4-mL samples) was collected in K_2_EDTA-coated Vacutainers (BD Diagnostics, Franklin Lakes, NJ, USA), a pre-dose sample was taken (time 0), while other samples were taken at 0.25, 0.5, 0.75, 1, 1.5, 2, 2.5, 3, 3.5, 4, 5, 6, 8, 12, 24, 36 and 48 h after drug administration. Cells and plasma were separated (10 min at 1600 × *g* at 4 °C). Plasma was stored at −65 ± 15 °C until use while DNA was extracted from cells.

### Plasma ATV quantitation

Proteins were eliminated by acetonitrile precipitation. Briefly, 400 µL acetonitrile was added to a 100-µL plasma sample, vortexed (70 rpm, 4 min), and centrifuged (9600 × g, 10 min, 10 °C). The supernatant (300 µL) was injected into a high-performance liquid chromatography-tandem mass spectrometry equipped with an Agilent 1100 control system (Agilent Technologies, Inc., Santa Clara, CA, USA) using a method validated by Ipharma S.A^6^. that respects Mexican regulations^54^ and guidelines for the validation of bioanalytical methods of the European Medicines Agency (EMA)^55^. An electrospray ionization tandem mass spectrometry system with a precursor ion (+) 559.3 m/z and a product ion (+) 440.3 m/z was used for detection. The linearity of the analytical method was assessed with calibration curves (0.5, 2.5, 5, 10, 25, 50 and 100 ng/mL), which yielded a correlation coefficient (r) = 0.99292 and a lower limit of quantification (LLOQ) = 0.5 ng/mL of ATV in plasma. Precision was assessed with control samples (1.7, 7.5, 35, and 75 ng/mL) and was considered within an acceptable range: intraday CV < 5% and interday CV < 8%. An accuracy error ≤7% was considered to be acceptable. The recovery was 87% and no effects were detected for the matrix, hemolyzed, and lipemic plasma (CV < 4%).

### Pharmacokinetic analysis

The C_max_ and time to reach C_max_ (T_max_) were obtained from the concentration-time data. The pharmacokinetics parameters were determined by non-compartmental methods. The AUC_0-t_ and AUC_0-∞_ were calculated with the log-linear trapezoidal rule. The Ke was estimated via log-linear regression from the terminal portion of the log-transformed concentration-time plots. T_1/2_ was estimated by dividing 0.693 by Ke. The Cl was calculated by dividing the dose by AUC_0-∞_ and adjusting for weight. The V_d_ was calculated as Cl divided by Ke. The AUC’s and C_max_ values were adjusted for dose and weight (AUC’s/dW and C_max_/dW)^17,56^. The pharmacokinetic analysis was performed using WinNonlin software v5.3 (Pharsight Corp., Mountain View, CA, USA).

### Determination of ATV metabolizer phenotypes

To estimate the ideal number of phenotypes, we applied a modified four-step multivariate analysis of the combined dose-and-weight-adjusted pharmacokinetic parameters C_max_, AUC_0-t_, AUC_0-∞_ and Cl^6,14,16,57^. First, hierarchical cluster analysis (HCA) was performed with the Ward linkage method and the distance matrix was calculated with the Manhattan measure. These analyses were applied to z-score transformed pharmacokinetic values to circumvent the comparability problems caused by the different scales of the non-transformed pharmacokinetic values. Aforementioned analysis were carried out with Minitab 16 software (Minitab Inc., State College, PA, USA). Second, we identified the subjects in each cluster, calculated the means of the adjusted pharmacokinetic parameters, and assigned the clusters to metabolizer phenotypes based on the means of the pharmacokinetic parameters. Third, we validated the phenotyping model by automatic linear modeling with the forward stepwise method, the Akaike Information Criterion (AICC) and the Overfit Prevention Criterion (ASE); followed by linear and logistic regression analysis. Finally, the cut-off limits were assessed by one-way analysis of variance and the Kruskal-Wallis test, considering *P* < 0.05 to be statistically significant different, with SPSS for Windows V.25 (IBM Corp., NY, USA).

### Genotyping tests

Genomic DNA that had been isolated with the alkaline lysis method^58^ was quantified by fluorometry using the Qubit dsDNA BR assay kit and a Qubit 3.0 fluorometer (Invitrogen; Thermo Fisher Scientific, Waltham, MA, USA). Compliance with DNA purity (OD_260_/OD_280_ between 1.8 and 2) was assessed with a Nanodrop 1000 spectrophotometer (Thermo Fisher Scientific, Waltham, MA, USA). DNA was stored at 10 ng/µL at −20 °C until analysis.

The polymorphisms *ABCB1*-rs1045642, *SLCO1B1*-rs4149056, *CYP2B6*-rs3745274, *CYP2D6*-rs16947, *CYP2D6*-rs1135840, *NAT2*-rs1208 and *COMT*-rs4680 were genotyped using a QuantStudio 1 real-time PCR system and TaqMan genotyping assays (Applied Biosystems; Thermo Fisher Scientific, Wilmington, MA, USA) according to the manufacturer’s protocol. Briefly, the PCR was prepared with 1X TaqMan Universal PCR Master Mix (Thermo Fisher Scientific, Inc.), 1X TaqMan genotyping assay mix, 10 ng DNA, and nuclease-free water to a total volume of 10 µl. Thermal cycling conditions were as follows: 95 °C for 10 min, 40 cycles of 95 °C for 15 sec and 60 °C for 1 min. Quality controls included a genotyping control using previously genotyped samples across different platforms, genotype call rate equal to 1.0, a Hardy-Weinberg equilibrium test with *P* > 0.05, and a minor allele frequency> 0.01. To detect the combination of the most important variants in the study population, a haplotype analysis was performed using the Haplotype Analysis Software V.1.05^59^, under a modified data entry method for diploid genomes.

### Statistical analysis

The sample size calculation considered an intrasubject coefficient of variation (CV) of 45% for C_max_ and AUC, a confidence interval (CI) of 90%, a significance level of 5%, a minimum power of 80%, and a Ω of 0.25. Thus, a sample size of 58 would suffice. The Hardy-Weinberg equilibrium was determined by comparing the genotype frequencies with the expected values using the maximum likelihood method^60^. To assess the effects of polymorphisms on the ATV pharmacokinetic parameters, comparisons between two and three groups were made. The Student’s *t*-test and one-way analysis of variance were used for parametric distributions, while Mann-Whitney U and Kruskal-Wallis H tests were used for nonparametric distributions. Post hoc tests (Bonferroni’s and Tamhane’s T2) were used for pairwise comparisons. To confirm the contribution of genetic factors to the variability of pharmacokinetic parameters, automatic linear modeling with the forward stepwise method, AICC and ASE was performed, as well as linear regression analysis with various modes (default mode, stepwise, remove, backward and forward). Possible associations of genotypes or combinations of genotypes with phenotypes were evaluated using X^2^ and Fisher’s exact tests and validated by logistic regression analysis. The evaluation effect of polymorphisms and associations were assessed under four different models (co-dominant, dominant, over-dominant and recessive). The odds ratio (OR) was estimated with a 95% CI. All *P* values were two-tailed. Corrected *P* values (Pc) were obtained using the Bonferroni correction for exclusion of spurious associations. *P* < 0.05 was interpreted as statistically significant. The statistical analyses were performed with SPSS for Windows, V.25 (IBM Corp., NY, USA).

## Data Availability

All data generated and analyzed during this study are included in this published article, but if necessary, some additional information is available from the corresponding author on reasonable request.
